# Model fidelity of group antenatal and postnatal care: a process analysis of the first implementation of this innovative service model by the Preterm Birth Initiative-Rwanda

**DOI:** 10.12688/gatesopenres.13090.1

**Published:** 2020-01-07

**Authors:** Elizabeth Butrick, Tiffany Lundeen, Beth S. Phillips, Olive Tengera, Antoinette Kambogo, Yvonne Delphine Nsaba Uwera, Angele Musabyimana, Felix Sayinzoga, David Nzeyimana, Nathalie Murindahabi, Sabine Musange, Dilys Walker

**Affiliations:** 1 Institute for Global Health Sciences, University of California San Francisco, San Franciso, CA, 94158, USA; 2 School of Nursing and Midwifery, National University of Rwanda, Kigali, Rwanda; 3 Rwanda Nurses and Midwives Union, Kigali, Rwanda; 4 School of Public Health, National University of Rwanda, Kigali, Rwanda; 5 Maternal, Child and Community Health Division, Rwanda Biomedical Center, Kigali, Rwanda

**Keywords:** Antenatal care, postnatal care, sub-Saharan Africa, group care, group prenatal care

## Abstract

**Background:** For a large trial of the effect of group antenatal care on perinatal outcomes in Rwanda, a Technical Working Group customized the group care model for implementation in this context. This process analysis aimed to understand the degree of fidelity with which the group antenatal care model was implemented during the trial period.

**Methods:** We used two discreet questionnaires to collect data from two groups about the fidelity with which the group antenatal care model was implemented during this trial period. Group care facilitators recorded descriptive data about each visit and self-assessed process fidelity with a series of yes/no checkboxes. Master Trainers assessed process fidelity with an 11-item tool using a 5-point scale of 0 (worst) to 4 (best).

**Results:** We analyzed 2763 questionnaires completed by group care facilitators that documented discreet group visits among pregnant and postnatal women and 140 questionnaires completed by Master Trainers during supervision visits. Data recorded by both groups was available for 84 group care visits, and we compared these assessments by visit. Approximately 80% of all group visits were provided as intended, with respect to both objective measures (e.g. group size) and process fidelity. We did not find reliable correlations between conceptually-related items scored by Master Trainers and self-assessment data reported by group visit facilitators.

**Conclusions:** We recommend both the continued participation of expert observers at new and existing group care sites and ongoing self-assessment by group care facilitators. Finally, we present two abbreviated assessment tools developed by a Rwanda-specific Technical Working Group that reviewed these research results.

## Introduction

This article reports process results associated with a study of group antenatal and postnatal care (ANC and PNC) conducted by the Preterm Birth Initiative-(PTBi) Rwanda in 2017–2019 ( Musange *et al.,* 2019). In the parent study, 18 Rwandan health centers were randomized to provide group ANC and PNC instead of the standard individual ANC and PNC, while 18 pair-matched health centers continued to provide individual ANC and PNC. The primary outcome of the parent study is gestational length; that analysis will be reported separately. This nested study examines the model fidelity of group ANC and PNC implemented for the purpose of this trial and the implications of our results on scaling implementation of group ANC and PNC in Rwanda and in other contexts.

### What is group care?

Group ANC and PNC visits include facilitated group discussion among a semi-consistent cohort of women and clinical care providers as well as indicated health assessments cooperatively conducted by group members in the same space where discussion occurs ( Rising & Quimby, 2016). These group visits serve all the purposes of routine ANC and PNC, and multiple studies report that group health care offers increased opportunities for health literacy, stress management, and health-promoting choices among participants ( Catling *et al.,* 2015; Felder *et al.,* 2017; Mazzoni & Carter, 2017; Patil *et al.,* 2017).

The foundational concepts of group ANC and PNC include cooperation and the dissolution of hierarchies, adult learning theory, and relationship-centered health care ( Manant & Dodgson, 2011; Novick *et al.,* 2013; Rising, 1998).

### Group ANC and model fidelity measurement

While numerous studies report individual outcomes after group ANC participation, only one published report of model fidelity measurement and outcome associations is available. A nested study of the effects of model fidelity on outcomes was performed within a parent study of group ANC conducted among young, low-income women in New York City; that parent study reported significantly decreased rates of preterm birth, adequate ANC attendance, and increased breastfeeding initiation among group ANC participants compared to individual ANC participants ( Ickovics *et al.,* 2007). The associated study of model fidelity measured group care process using two items scored by an observer on a scale from 1 to 10: 1) “To what extent was the group session didactic vs. facilitative?” and 2) “How much were group members involved and connected?” Researchers assessed content fidelity (23 discussions topics planned over 10 group ANC visits) by calculating the proportion of recommended topics for the group visit that were actually discussed, as reported by group care providers. Researchers found that greater process fidelity was associated with lower odds of preterm birth among participants, while greater content fidelity was not associated with a lower odds of preterm birth ( Novick *et al.,* 2013).

### Group ANC and PNC in Rwanda

In Rwanda, routine ANC is offered only at the health center level and is most commonly provided by a nurse ( National Institute of Statistics of Rwanda, Ministry of Finance and Economic Planning, Ministry of Health, 2016). The group care model was customized for implementation in health centers by local stakeholders; that process and the resulting model including content are described in a separate publication ( Sayinzoga *et al.,* 2018). In summary, the group care package implemented for the parent trial includes the initial ANC visit completed individually with a provider, 3 group ANC visits, 3 individual PNC visits before 6 weeks after birth, and a group PNC visit at approximately 6 weeks after birth. All the fundamental components of the group care model as described by Rising and Quimby ( Rising & Quimby, 2016) were prioritized in this implementation, and each group visit was meant to be co-facilitated by one ANC provider (either a nurse or midwife) and one community health worker (CHW).

The Preterm Birth Initiative-Rwanda trial of group ANC/PNC is a cluster randomized controlled trial powered to detect a 0.5-week increase in gestational length among women who received care at 18 of 36 facilities randomized to group care. As such, the sample size of women eligible for analysis is large (over 11,000 women and their newborns). We estimated that about 200 group visits per month would be convened at all 18 group care study sites during the trial period. In this process analysis, we aimed to understand the degree of fidelity with which the group ANC/PNC model, as defined by the Rwanda group ANC/PNC Technical Working Group, was implemented during this trial period. We also aimed to use this data set to make recommendations to the Rwanda Ministry of Health regarding group care model fidelity monitoring after this trial.

## Methods

A total of 5/30 Rwandan districts were selected for the parent trial in collaboration with the Rwanda Ministry of Health. Within those 5 districts, 55 health centers were assessed with a standardized tool for number of providers, ANC volume, suitable space for group care, services, and equipment. Health centers that reported that they allocate at least two providers to ANC services on any day that ANC is offered were selected for this trial, for a total of 36 health centers. These 36 health centers were pair-matched and then randomized to either continue individual ANC and PNC or switch to group ANC and PNC. A data collector was embedded at each of the 36 study sites.

To both monitor and study model fidelity during the trial, the study partners developed a strategy to: 1) train Rwandan group ANC/PNC Master Trainers who in turn trained ANC/PNC providers to deliver the group care model; 2) provide regular support and feedback to group care providers by Master Trainers who visited and observed group visits at all intervention clinics; 3) collect model fidelity data by Master Trainers during observed group visits; and 4) instruct group care providers to complete a self-assessment instrument after each group visit, observed or unobserved by the Master Trainer.

This analysis includes data collected by two groups between late June 2017 and early January 2019. The first group includes nurses, midwives, and CHWs who were recruited by facility directors to participate in the PTBi-Rwanda trial as group ANC and PNC facilitators. The second group includes one nurse, five midwives, and one physician who served as group ANC/PNC Master Trainers; the preparation of these Master Trainers is described elsewhere ( Sayinzoga *et al.,* 2018). We used two discreet questionnaires to collect data from these two groups about the fidelity with which the group ANC/PNC model was implemented during this trial.

### Participant recruitment and data collection process

***Group Visit Debrief Questionnaire.*** Shortly before the PTBi-Rwanda trial began, providers and CHWs selected to be group care facilitators were invited to attend one of several three-day training meetings. At the close of this training meeting, providers and CHWs were invited to participate in the trial as group care facilitators and as research subjects. As research subjects, they were instructed to complete a Group Visit Debrief Questionnaire (GVDQ) after each group visit they facilitated, and they consented to collection and analysis of the data reported in these questionnaires. Each provider or CHW who consented was assigned a unique, 5-digit identification (ID) code, and questionnaires recorded the ID codes of all participating facilitators.

Data was collected through one of three methods: 1) the facility-embedded data collector used a tablet to collect data from facilitators after the group visit and later synced the tablet’s data to an electronic data capture system ( Harris *et al.,* 2009); 2) facilitators entered the data themselves on the tablet; or 3) facilitators completed a paper version of the questionnaire when the tablet was not available, and this data was entered later into the tablet by the data collector. Questions and answers were provided in Kinyarwanda, the language used among providers, CHWs, and women during group care. Completing this questionnaire required about 10 minutes and aimed to inspire discussion among co-facilitators about the quality of each group visit they conducted.

***Model Fidelity Assessment.*** Providers and CHWs were also asked to consent to be observed by Master Trainers during future group ANC/PNC visits and to allow for data collection during those observations. With each observation the Master Trainer documented the quality of the group visit using a Model Fidelity Assessment (MFA) tool. Co-facilitators’ unique study IDs were recorded in the MFA tool.

Master Trainer visits to each of the 18 health centers were scheduled at months 1, 2, 3, 5, 7, 9, 12, 15, and 18 after the introduction of group care. Some health centers received additional visits if they asked for help or if the Master Trainers noted that the facilitators needed additional coaching. An MFA was completed each time a group visit was observed. In a few cases, a group visit did not occur as planned after the Master Trainer arrived at the health center—sometimes group visits were cancelled if fewer than four women attended, and occasionally health center staff miscommunicated with Master Trainers about the dates on which group visits were scheduled. In these cases, Master Trainers offered support and mentorship but did not complete an MFA. The MFA required about 10 minutes to complete, but each Master Trainer visit lasted between 4–8 hours as the visit was also intended to provide intensive support and coaching for the facilitators and directors of health centers.

### Data collection tools

We created two tools to monitor implementation and fidelity ( *Extended data*; Lundeen *et al.,* 2019). We did not validate the tools prior to implementation.

***Group Visit Debrief Questionnaire.*** The GVDQ includes basic descriptive data about each visit, including date, number and titles of co-facilitators, number of pregnant or postnatal women in attendance, and time spent on group care activities. Additionally, the study team hoped to inspire co-facilitators to discuss the successes and challenges of the group visit by including three process questions: 1) What went well today? 2) What didn’t go as well as we had hoped? and 3) What can we do to ensure that the next group visit is even better than this one? Group care facilitators could choose pre-determined answer choices and/or free text answer options for each of these questions. The pre-determined “check-box” answers were meant to remind facilitators of both positive and negative process indicators upon which they could reflect as a semi-structured, recurring learning and problem-solving activity. A checked box was considered a “yes” answer and a blank box was considered a “no” answer.

***Model Fidelity Assessment.*** Master Trainers completed a 12-item questionnaire after each group ANC or PNC visit they observed. This instrument was created collaboratively by members of the Group ANC/PNC Technical Working Group, group care Master Trainers and the group ANC technical advisor based at UCSF. Referring to the published literature about group ANC and prioritizing coherence with the Rwanda ANC and PNC service packages, we decided on 12 items to measure group visit model fidelity using a 5-point Likert scale, from a minimum score of 0 (“facilitators could not perform even though the opportunity was present”) to a maximum score of 4 (“facilitators were fully competent”) for the MFA.

### Analysis and interpretation

***Group Visit Debrief Questionnaire.*** Quantitative data from the GVDQ were analyzed with linear and multiple regression analyses to compare: 1) overall MFA scores to data collected in the GVDQs, and 2) individual MFA items to individual GVDQ items that are conceptually related.

***Model Fidelity Assessment.*** Each of the 12 items of the MFA was scored with a number between 0 and 4, according to a scoring rubric. We removed one item from this tool before analysis. The item “Husbands and next-of-kin were engaged and participated in activities (if they were present)” was scored in only 7% of MFAs, so we removed it for the purposes of this analysis. We created an overall MFA score for each observed visit by finding the average of the remaining 11 individual item scores by session. We used summary statistics to understand the results of all available MFAs completed by Master Trainers and the GVDQs completed by group care facilitators. We then used STATA to perform linear and multiple regressions to compare: 1) overall MFA scores to GVDQ score and individual items and 2) individual MFA items to individual GVDQ items that are conceptually related. Following this initial analysis, we then matched group visits for which an MFA and a GVDQ were both available, in order to compare data provided by Master Trainers and group visit co-facilitators corresponding to the same group visit. We used linear regression to determine which descriptive visit characteristics from the GVDQ and/or MFA items were most closely correlated with the average MFA score, using data from this sub-set of observed group visits. We also compared answers to conceptually-related questions from these two tools, to discover how often the answers agreed.

## Results

Of about 3000 expected group visits, 2763 total GVDQs were available (approximately 90%); 17 records were excluded for missing or nonsensical data. Of 162 expected Master Trainer observations, 149 MFAs were available, but 9 of these were missing data for one or more items and 140 were analyzed. Both GVDQs and MFAs were available for 84 visits. Table 1 reports documentation of group ANC and PNC visits recorded by facilitators in the GVDQ, both during the 84 visits for which MFAs were also available and for all documented group visits (2763). Of note, women required some amount of additional, one-on-one care during or after about one-third of group visits, and the average number of women who required this individual care was one woman per group. Co-facilitators wrote in descriptions of this additional, one-on-one care for only about 20% of these (n=175). In total, 87% of these free-text responses stated that the additional care was for preventative services including provision of a family planning method (after PNC), provision of an insecticide-treated bed net, and tetanus toxoid vaccination. A total of 13% these responses documented the management of abnormal findings, including symptoms of malaria, abnormal health assessment findings, and transfer to hospital.

**Table 1.** Characteristics of group visits. Group Visit Debrief Questionnaire results from all documented visits and a sub-set of visits observed by a Master Trainer. CHW, community health worker; NA, not applicable.

Descriptive characteristic	Observed by Master Trainers (n=84)			All (n=2763)		
	Average	IQR	Range	Average	IQR	Range
Number of pregnant or postnatal women who participated in the group visit	9	7-11	3-22	9	7-11	2-27
Number of minutes spent in health assessments	56	30-68	10-240	51	38-60	8-240
Number of minutes spent in group discussion	69	59-83	30-122	63	51-70	30-167
Total number of minutes spent, group visit including health assessment and group discussion	126	100-150	38-294	114	96-126	40-294
Number of minutes spent during group visit, divided by the number of women in attendance	13	10-18	4-32	12	10-16	3-63
In those groups in which at least one woman needed one-on-one care, number of women who needed this additional care after group visit	2	NA	1-5	1	NA	0-10
Average number of minutes required to provide this additional one-on-one care for all women who required it	17	6-20	4-60	19	10-23	2-120
	**Observed by Master** **Trainers (n=84), %**			**All (n=2763) Proportion**		
Group visit was co-facilitated by at least one provider and one CHW	80			89%		
Group visit facilitated by one or more CHWs, without documentation of a provider in attendance	3			2%		
At least one midwife in attendance as a co-facilitator	19			30%		
Yes, we provided clean water for the group visit participants to drink	85			83%		
Group visits in which some woman participants were “drop-ins”—that is, they were not regular, assigned members of the group	48			61%		
In those groups in which some participants were “drop-ins,” % of women who were drop-ins	29			20%		
Visits attended by at least one male partner	4					
Visits attended by at least one female guest (“next of kin”)	3					
Group visits in which at least one woman needed additional, one-on-one care during or after the group visit	50					

Table 2 reports self-assessment items in the GVDQ, among both all documented group visits and the sub-set of visits observed by a Master Trainer. In general, group visit facilitators self-reported positive characteristics of the visit more often among all visits than among the sub-set of visits observed by Master Trainers. The mean score for all 140 complete MFAs was 3.17 (5-point scale from 0-4). The mean MFA score among the sub-set of records with an available GVDQ (n=84), was 3.16. (1.09 to 4.0). The average visit score among visits at which a midwife was present (19% of visits) was slightly higher than visits at which a midwife was not present (81% of visits), but this difference was not statistically significant. There was a range of mean score calculated by health center, from 2.95 to 3.50. The trend line for the average score increased approximately 13% from the beginning to end of this 18-month period. We compared MFA score with 6 characteristics of each group visit: 1) the health center where the group visit was observed, 2) whether or not water was prepared for the women’s refreshment, 3) the title of the facilitator present with the highest level of education (nurse or midwife), 4) the number of facilitators present, 5) the number of women present for the group visit, and 6) the length of time in days since the study began. There was a correlation between length of time since the study began and MFA score (F-score=0.0003, r2=0.15). However, there were no significant correlations between the MFA score and any of the other characteristics listed above.

**Table 2.** Group Visit Debrief Questionnaire, subjective self-assessment items.

What went well?	Yes responses (%); observed visits = 84	Yes responses (%); documented responses = 2763
Group participants were all engaged and participated in activities	88	88
The group demonstrated trust and unity	86	91
Participants spoke more than co-facilitators spoke during the group discussion today	80	92
All participants understood the information we discussed	84	91
We were well organized	81	87
We worked well together as a team	71	81
We followed the lead of the women	58	82
We provided all the assessments, treatments, and referrals required by the women present today	51	68
We kept time	40	76
Husbands and/or next-of-kin, if present, were engaged in activities	7	8

Table 3 shows the average MFA score for each item, across all 140 complete MFA records. The highest average score (3.46) for a single item was “The co-facilitators provided ANC/PNC screening, medications, and referrals as indicated, consistent with the Rwanda FANC and PNC packages,” indicating that the overall quality of service package delivery was high. The item with the lowest average score was “Kept time,” (2.70) which is consistent with the result reported in Table 1 that at least 25% of group care visits lasted more than 2 hours. Table 4 shows the correlation scores resulting from bivariate regression analysis comparing each of the 11 MFA items to the overall MFA score. Four MFA items had correlation scores between 0.71 and 0.76 (highest correlation score), while 7 MFA items had correlation scores between 0.45 and 0.69. Several self-reported items in the GVDQ are conceptually closely related to items in the MFA; these conceptually-related items appear in Table 5. We performed a bivariate regression analysis with the sub-set of records with data from both tools (n=84) to discover whether a “yes” answer to each of this sub-set of items in the GVDQ could predict whether the related item in the MFA would be scored 3 or 4 (0-4 scale with 4 being the highest possible item score) by the Master Trainer. There was not a strong relationship between self-reported and Master Trainer scores for any of these conceptually-related items; self-assessments both over- and under-reported soft skills compared to Master Trainer assessments. However, two GVDQ items agreed with MFA items ≥68% of the time: we were well-organized and we followed the lead of the women.

**Table 3.** Average MFA score, by item (scale 0-4 *), among 140 MFA records. MFA, Model Fidelity Assessment.

MFA item	Average score
The co-facilitators provided ANC/PNC screening, medications and referrals as indicated, consistent with the Rwanda FANC and PNC packages	3.46
The co-facilitators performed assessments correctly and followed up on abnormal findings	3.42
The co-facilitators communicated using language well understood by all participants, and responded appropriately to verbal and non-verbal cues	3.35
The co-facilitators followed the lead of the women and could flexibly adjust the visit agenda to better meet women's needs and interests	3.33
The co-facilitators encouraged active participation in group activities/discussions and payed particular attention to participants who presented as reserved	3.18
The co-facilitators demonstrated mastery (accurate knowledge) of the curriculum, including discussion topics and key messages	3.13
Participants spoke more than the co-facilitators spoke	3.13
The co-facilitators prepared the group care room environment, including assessment equipment, learning materials, participant refreshment, and indicated medications	3.09
The co-facilitators reinforced individual and group accomplishments	3.03
The co-facilitators kept time	2.70

* 0=Facilitators could not perform this skill even though the opportunity was present; 1=Facilitators made attempts but needed significant help and to be retrained.

**Table 4.** Correlation scores: relationship between each of 11 MFA items and overall MFA score (n=140). MFA, Model Fidelity Assessment.

MFA item	Correlation score
*The co-facilitators:*	
Demonstrated mastery (accurate knowledge) of the curriculum, including discussion topics and key messages	.76
Followed the lead of the women and could flexibly adjust the visit agenda to better meet women's needs and interests	.76
Reinforced individual and group accomplishments	.74
Prepared the group care room environment, including assessment equipment, learning materials, participant refreshment, and indicated medications	.71
Performed assessments correctly and followed up on abnormal findings	.69
Communicated using language well understood by all participants, and responded appropriately to verbal and non-verbal cues	.66
Provided ANC/PNC screening, medications and referrals as indicated, consistent with the Rwanda FANC and PNC packages	.60
Encouraged active participation in group activities/discussions and payed particular attention to participants who presented as reserved	.59
Kept time	.58
Asked open-ended questions to promote discussion	.57
Ensured that participants spoke more than the co-facilitators spoke	.45

**Table 5.** Conceptually-related items in the Group Visit Debrief Questionnaire and the Model Fidelity Assessment and agreement between these items across tools.

Model Fidelity Assessment Item (Master Trainer scored this 3 or 4 on a scale of 0-4)	Group Visit Debrief Questionnaire Item (Facilitators answered “yes” for this item)	Agreement between scoring of these two items for the same visit (%)
The co-facilitators demonstrated mastery (accurate knowledge) of the curriculum, including discussion topics and key messages	We were well organized	76
During the group care visit today, the co-facilitators: Prepared the group care room environment, including assessment equipment, learning materials, participant refreshment, and indicated medications	We were well organized	75
Followed the lead of the women and could flexibly adjust the visit agenda to better meet women's needs and interests	We followed the lead of the women	68
Performed assessments correctly and followed up on abnormal findings	We provided all the assessments, treatments, and referrals required by the women present	53
Communicated using language well understood by all participants, and responded appropriately to verbal and non-verbal cues	All participants understood the information we discussed	76
Encouraged active participation in group activities/ discussions and payed particular attention to participants who presented as reserved	Group participants were all engaged and participated in activities	77
Kept time	We kept time	47
Participants spoke more than the co-facilitators spoke	Participants spoke more than co-facilitators spoke	78

## Discussion

Our results are consistent with previously reported results of group ANC model fidelity by Novick *et al.* in the United States ( Novick *et al.,* 2013). Those authors reported that fidelity to intended group ANC process was 77% (range, 54–97%) and intended group ANC content was 70% (range, 44–100%). We did not specifically measure content fidelity, but overall model fidelity—focused on process fidelity—during this study period was estimated to be 80% (average MFA score was 3.18 on a 0-4 scale). The Master Trainers observed that, in general, this cohort of group ANC and PNC adhered to the Rwanda package of ANC/PNC services while implementing this alternative model of service delivery. By objective descriptive data, the intervention was implemented as intended. The “soft” skills fundamental to the success of the group care intervention were challenging to learn and implement (as expected), but Master Trainers observed that, across this study period, providers encouraged participant engagement, asked open-ended questions, and spoke less than participants during discussions to a degree estimated to be, on average, 80% of the ideal. However, when co-facilitators rated themselves on these soft skills, their answers did not consistently agree with Master Trainer assessments. Self-assessment of these soft skills may be less discriminating than Master Trainer assessment of these important facilitative leadership skills.

Model fidelity scores were not significantly different depending on whether a nurse or a midwife was the “highest-level” provider present at the group visit. We interpret this result to mean that in this context nurses and midwives are equally able to successfully provide group care. The presence of 1, 2, or 3 facilitators also had no relationship to the MFA score. We interpret this to mean that while 2 co-facilitators may make it easier to share the labor of providing the group visit, 1 provider is equally as likely as 2 or more co-facilitators to achieve a high MFA score. Because CHWs in Rwanda do not independently complete blood pressure and abdominal examination at this time, we assume that they cannot independently convene a group visit that is meant to include these assessments and clinical decision-making for abnormal findings.

In future implementation of group ANC and/or PNC care in Rwanda, we recommend two abbreviated group ANC/PNC model fidelity assessment tools, one for facilitators and one for expert observers ( Table 6 and Table 7). These abbreviated tools were created by the Rwanda Group ANC/PNC Technical Working Group after reviewing our results. The Rwanda Group ANC/PNC Technical Working Group’s activities and composition are described in a separate publication ( Sayinzoga *et al.,* 2018). These simplified assessment questionnaires could be integrated into a streamlined monitoring strategy. We conclude that future implementation of group ANC/PNC in Rwanda will benefit from continued collection of self-assessment data by group visit facilitators, expert coaching and mentoring, and assessment by expert observers. Future research is needed to understand the optimal schedule of observation visits by quality assurance supervisors.

**Table 6.** Group ANC/PNC model fidelity assessment, completed by facilitator(s). ANC, antenatal care; PNC, postnatal care; CHW, community health worker.

Data item	Answer (circle one or write in answer)
Date of group visit
Start time and end time
Which group visit was it? (which GANC visit or which GPNC visit)
Number of CHWs in attendance
Number of women in attendance
Water available for women to drink	Yes No
Room prepared with chairs in circle	Yes No
Private area with screen used for health assessments BP machine and weighing scale used	Yes No
Materials used for planned discussion topics	Yes No
Topics discussed during this visit	
We followed the lead of the women	Yes No

**Table 7.** Group ANC/PNC model fidelity assessment, completed by expert observer(s). ANC, antenatal care; PNC, postnatal care; CHW, community health worker.

Data item	Answer (circle one or write in answer)
Date of group visit		
Which group visit was it? (which GANC visit or which GPNC visit)		
Number of providers in attendance		
Number of CHWs in attendance		
Number of women in attendance		
Did the co-facilitators demonstrate mastery (accurate knowledge) of the curriculum, including discussion topics and key messages?	Yes No
Did the facilitators let women speak more?	Yes No
Did the facilitators follow the lead of the women?	Yes No

While about 25% of group visits lasted longer than 2 hours, we found that it is feasible in this context to plan for three group ANC and one group PNC visits that last an average of 2 hours with an average of nine women in attendance. These results give Rwandan policy makers information when they consider whether patient volumes and human resources necessary for future implementation of group ANC and PNC are aligned. For example, a health center that provides comprehensive ANC for an average of 50 women who expect to give birth every month, and that invites all pregnant women to three follow-up group ANC visits over the course of pregnancy, should plan for about 40 hours per week of work dedicated exclusively to group ANC provision.

### Limitations

We acknowledge that this process analysis had some significant limitations. First, GVDQs were missing for 38% of visits that were observed by Master Trainers. Providers may have skipped the completion of the Debrief instrument when they engaged in a discussion with the Master Trainer after the observed group visit. By comparing the number of women enrolled in the trial to the number of woman participants documented in the GVDQs, we estimate that less than 5% of group visits are missing from our data set. Second, Master Trainer visits were conducted by seven different individuals and we did not assess inter-rater reliability for the MFA, due to financial and logistical limitations. Ideally, the group ANC/PNC technical advisor would have independently scored each the MFA during each observation visit to be able to later compare inter-rater scores. Finally, this analysis assumes that the Model Fidelity Assessment score is the “gold standard” by which we should evaluate the degree to which women received group ANC/PNC during the trial period. While we are confident in the expertise and understanding of the Master Trainers, there may be more comprehensive methods to monitor model fidelity that integrate both expert observer and self-assessment measurement into a single tool or internally consistent set of tools. Ideally, model fidelity measurement would integrate quantitative and qualitative feedback from women to provide a more complete assessment that includes participant, facilitator, and expert observer experiences of each group visit.

## Conclusion

Our findings from the first implementation of group ANC and PNC in Rwanda suggest that the model was delivered as intended during the majority of group visits, with evidence from both self-assessments and expert observations. However, we did not find significant correlations between MFA scores provided by Master Trainers and self-assessment data reported by group visit facilitators. At this time and in this context, we cannot recommend relying on facilitator self- assessment alone to monitor group ANC/PNC process fidelity. We recommend both the continued participation of expert observers at new and existing group care sites and the ongoing development of self-assessment techniques that may lead to more reliable self-monitoring methods for even larger-scale group ANC/PNC programs.

## Ethical statement

Ethical approval for all study activities, including the administration of these two questionnaires, was granted by the Rwanda National Ethics Committee (0034/RNEC/2017) and University of California, San Francisco Institutional Review Board (16-21177). Two discreet written informed consent forms were obtained from each provider and CHW prior to the first group ANC or PNC visit in which she/he participated as a facilitator: one consent form for completing GVDQ and the other consent form for being observed by Master Trainers while facilitating a group ANC or PNC visit. No personal identifiers of providers or CHWs were recorded. Study staff protected all data as confidential.

## Data availability

### Underlying data

Dryad: Group antenatal and postnatal care in Rwanda: model fidelity monitoring data, https://doi.org/10.7272/Q6QN64X5 ( Lundeen *et al.,* 2019).

### Extended data

Dryad: Group antenatal and postnatal care in Rwanda: model fidelity monitoring data, https://doi.org/10.7272/Q6QN64X5 ( Lundeen *et al.,* 2019).

This project contains the following extended data:

- Group Visit Debrief Questionnaire
- Model Fidelity Assessment tool

Data are available under the terms of the Creative Commons Zero "No rights reserved" data waiver (CC0 1.0 Public domain dedication).
