# Supplementary material for: Model fidelity of group antenatal and postnatal care: a process analysis of the first implementation of this innovative service model by the Preterm Birth Initiative-Rwanda
Source: Gates Open Res. 2020 Jan 7;4:7. [Version 1] doi: 10.12688/gatesopenres.13090.1 (PMC7177154; doi:10.12688/gatesopenres.13090.1)
Supplement: Peer review history

# Peer review: Reviewer response for version 1

**DOI:** 10.21956/gatesopenres.14239.r28738

**Published:** 2020-04-22

**Authors:** Anthony Idowu Ajayi

**Affiliations:** 1 Population Dynamics and Sexual and Reproductive Health and Rights Unit, African Population and Health Research Center, Nairobi, Kenya

This is an interesting, important and well-written study.  The introduction provides the needed background and justification for the study. The methods described the study design, tools and analytical strategies. The results and discussions are easy to follow. The study limitations were highlighted.

 However, I have some questions. Is there is any reason for not validating the tools? Perhaps the authors could use PCA to assess the tools or at least estimate and Conbrach's Alpha coefficient to assess internal consistency. Also, I did not see the results of the linear and multiple regression analysis. Even though the discussion focused model fidelity, the authors may still comment on any aspect of the items in the tools return with a lower rating. E.g. kept time and Performed assessments correctly and followed up on abnormal findings.

 The conclusion is appropriate given the results presented.

I confirm that I have read this submission and believe that I have an appropriate level of expertise to confirm that it is of an acceptable scientific standard.

# Peer review: Reviewer response for version 1

**DOI:** 10.21956/gatesopenres.14239.r28427

**Published:** 2020-01-30

**Authors:** Sharon Schindler Rising, Deborah Billings

**Affiliations:** 1 Centering Healthcare Institute, Silver Spring, MD, USA; 2 Group Care Global, Silver Spring, MD, USA; 3 Institute for Families in Society Health Promotion, Education & Behavior, Arnold School of Public Health Columbia, Columbia, SC, USA; 4 Maternal and Child Health, Gillings School of Global Public Health, University of North Carolina at Chapel Hill, Chapel Hill, NC, USA

- The authors are to be commended for producing a careful analysis of the successes and challenges of implementing a group care model that changes the design of health care delivery. The article carefully builds on the published content of an earlier article (Sayinzoga *et al.*, 2018) that discusses their use of the facilitated process in the design and implementation of the group model. The reader is encouraged to access this article to more fully understand the complexity of the model.
- This model fidelity article describes data collected from group facilitators in 18 health centers and Master Trainers who observed the facilitators and provided consultation as needed. The article clearly underscores the trial’s involvement of the site partners in the conduct of the study. The bibliography provides additional resources for further understanding of the group care model used for the basic design of this Rwanda trial.
- We found an occasional term that was not defined clearly or whose definition was missing. For example, what are “conceptually-related items” and “soft items”? We also found terminology that seemed to be interchangeable, such as the use of “expert observers” and “Master Trainers,” but whose use was not clearly stated as being interchangeable.  
- We suggest discussion of why two different tools were used in the study. The complexity of the Master Trainer tool may make it too difficult for use outside of this large study.
- The following comments relate to points appearing on specific pages. When quotes are extracted from the manuscript, these are marked by quotation marks and are followed by reviewer comments in italics.

**Page 3:**

- “The foundational concepts of group ANC and PNC include cooperation and the dissolution of hierarchies, adult learning theory, and relationship-centered health care (Manant & Dodgson,
  2011; Novick *et al., *2013; Rising, 1998).”
*Add “facilitated” not didactic group dynamic as a foundational concept.*
- “While numerous studies report individual outcomes after group ANC participation, only one published report of model fidelity measurement and outcome associations is available” (cite reference: Novick *et al.*, 2013).

 

**Page 5:**

- Table 1 refers to IQR - please spell this out. This is the only time the term “IQR” is used.
- The Table 1 title seems to be missing a word. We suggest revising it to state “Group Visit Debrief Questionnaire from all documented visits (n=2763) and a sub-set of visits observed by a Master Trainer. Put “CHW, community health worker; NA, not applicable” as a table footnote. Having them in the title itself is confusing. Rearrange the columns so that the “All” categories columns come after the descriptive characteristics and the Observed by Master Trainer columns are at the right-hand end of the table.

**Page 8:**

- “Self-assessment of these soft skills may be less discriminating than Master Trainer assessment of these important facilitative leadership skills.” 
*We suggest using more precise language than “less discriminating” so that it’s clear that self-assessment resulted in higher scores of soft skills than those given by Master Trainers.*
- “Because CHWs in Rwanda do not independently complete blood pressure and abdominal examination at this time, we assume that they cannot independently convene a group visit that is meant to include these assessments and clinical decision-making for abnormal findings.”
*This seems out of place in the context of discussing MFA scores. This would be better placed in a section that discusses the scopes of work of different providers and how they fit or don’t fit with facilitating groups independent of other providers. *

**Page 9:**

- Tables 6 and 7: it’s not clear why the category “facilitators let women speak more” does not appear on the self-assessment (as, for example, “I let women speak more”). Overall, the phrasing is slightly confusing - what is meant by “speak more”? Does it refer to women speaking more often than the facilitators (we suspect it does given the information presented in Table 3, but this should be made clear in the instrument itself).
- The authors note that they “did not find significant correlations between MFA scores provided by Master Trainers and self-assessment data reported by group visit facilitators.”
*Did the scores tend to skew in one particular direction e.g. self-assessment scores were consistently higher than those of Master Trainers? The trend is mentioned on page 5 (“*In general, group visit facilitators self-reported positive characteristics of the visit more often among all visits than among the sub-set of visits observed by Master Trainers.”) *but it doesn’t stand out. The trend should be reported elsewhere in the manuscript, including the Conclusions.*
- Having Master Trainers observing the group is likely to influence the group facilitators and possibly the group dynamics. Acknowledgement of this might help to explain the differences in the scoring on the data collection tools. Also, it would be a reasonable expectation that confidence and competence of the facilitators would increase as their experience increases. We don’t see any discussion of a general change in scores from early to later groups.
- Changing systems to support different care models is daunting. The data from the group antenatal/postpartal models continues to demonstrate improvement in health outcomes and satisfaction of the participants with receiving and providing care in this way. We applaud this careful implementation/model fidelity study and urge continued focus on ways to study fidelity using less resource.

We confirm that we have read this submission and believe that we have an appropriate level of expertise to confirm that it is of an acceptable scientific standard.
